# Cardiopulmonary dysfunction in perinatally HIV‐infected South African adolescents on antiretroviral therapy: baseline findings from the Cape Town Adolescent Antiretroviral Cohort

**DOI:** 10.1002/jia2.25340

**Published:** 2019-07-10

**Authors:** Leah N Githinji, Sana Mahtab, Liesl Zühlke, John Lawrenson, Landon Myer, Diane Gray, Heather Zar

**Affiliations:** ^1^ South Africa MRC unit on Child and Adolescent Health Department of Paediatrics and Child Health Red Cross War Memorial Children's Hospital University of Cape Town Cape Town South Africa; ^2^ Division of Paediatric Cardiology Department of Paediatrics and Child Health Red Cross War Memorial Children's Hospital University of Cape Town Cape Town South Africa; ^3^ Division of Cardiology Department of Medicine Groote Schuur Hospital Faculty of Health Sciences University of Cape Town Cape Town South Africa; ^4^ Department of Paediatrics and Child Health Stellenbosch University Matieland South Africa; ^5^ Division of Epidemiology & Biostatistics School of Public Health & Family Medicine University of Cape Town Cape Town South Africa

**Keywords:** perinatal HIV infection, antiretroviral therapy, cardiopulmonary dysfunction, adolescents, HIV care continuum, South Africa

## Abstract

**Introduction:**

Antiretroviral therapy (ART) has reduced morbidity and mortality in sub‐Saharan Africa, but the burden of coexistent cardiopulmonary disease in perinatally HIV‐positive adolescents on antiretroviral therapy (ART) has not been well described. The aim of this study was to investigate the prevalence and associations of cardiopulmonary dysfunction in adolescents with perinatally acquired HIV on ART.

**Methods:**

For this cross‐sectional analysis, 515 perinatally HIV‐positive adolescents ages 9 to 14 years on ART for at least six months, and a comparator group of 110 age‐matched HIV‐uninfected adolescents were tested between August 2013 and April 2015 using echocardiography, six‐minute walk test (6MWT) and spirometry. Those with either abnormal spirometry or abnormal 6MWT and any right or left systolic or diastolic dysfunction or abnormal mean pulmonary arterial pressure were considered as having impaired cardiopulmonary function. Logistic regression was used to investigate determinants of impaired cardiopulmonary function.

**Results:**

Overall, 474 adolescents with perinatally acquired HIV (mean [SD] age, 12 [1.6] years; median [IQR] ART duration, 7 [4.6 to 9.3] years; median [IQR] CD4 count, 712 [571 to 959] cell/mm^3^) and 109 HIV‐uninfected adolescents mean (SD) age 11.8 (1.8) years, had successful cardiac and lung function testing. Impaired cardiopulmonary function was detected in 13% of adolescents with perinatally acquired HIV and 8% of HIV‐uninfected adolescents, *p* = 0.136. Among adolescents with perinatally acquired HIV, those with low tricuspid annular plane systolic excursion (TAPSE) had significantly lower mean FEV_1_, 1.5 L versus 1.6 L, *p* = 0.011. Height (OR 0.7, 95%CI 0.5 to 0.9), body mass index (OR 0.7, 95%CI 0.5 to 0.9) and past pulmonary tuberculosis (OR 2.3, 95%CI 1.2 to 4.4) were significantly associated with a low cardiopulmonary function.

**Conclusions:**

Despite being on ART, cardiopulmonary dysfunction occurs in an appreciable proportion of perinatally HIV‐infected adolescents but no significant difference to uninfected controls. This finding requires further exploration. Factors associated with dysfunction may be amenable to public health interventions to reduce cardiopulmonary disease in this population.

## 1 Introduction

With improved survival of adolescents with perinatally acquired HIV on antiretroviral therapy (ART), HIV has become a chronic disease, with perinatally infected children surviving into adolescence and adulthood 1. HIV‐related cardiac disease 2, 3, 4 and/or chronic lung disease has been reported in sub‐Saharan Africa 5, 6. Symptoms which can be attributed to either cardiac or lung disease like tachypnoea or dyspnoea can overlap posing a diagnostic challenge.

In the pre‐ART era, a high prevalence of cardiac abnormalities especially left ventricular dysfunction and cardiomyopathy were reported 7, but a sharp decline has been found with the use of ART 8, 9. Although ART has reduced the incidence and severity of acute pulmonary infections, lung function abnormalities are still highly prevalent 10, 11. Right heart dysfunction may be secondary to chronic lung disease. Miller *et al*. 4 reported a 29% prevalence of right ventricle dilatation in a Zimbabwe cohort of perinatally HIV‐positive adolescents (71% on ART, median duration of ART 20 months). Nearly 50% of this population had chronic lung disease.

ART also has an impact on exercise tolerance, which is a marker of cardiopulmonary function status. HIV‐positive children on ART in Malawi had better exercise tolerance compared with HIV‐positive ART‐naive children 12. ART‐naive HIV‐positive children had worse symptoms of cough, dyspnoea, hypoxaemia and low exercise tolerance compared with those on ART 13. Although ART is reported to improve cardiac function, exercise tolerance and lung function, children on ART still have lower lung function 11, lower exercise tolerance 12 and lower cardiac function 14 compared to HIV‐uninfected children. Most of these published studies were from the era when ART access was not universal, was initiated based on clinical and immunological severity; hence the need for studies from well‐established ART cohorts.

Risk factors for cardiopulmonary dysfunction may include HIV immunosuppression, opportunistic infections, poor ART adherence, late age at initiation of ART, malnutrition or smoking. Other risk factors may be specific to cardiac dysfunction like dyslipidaemia. Furthermore, transplacental exposure to drugs like zidovudine has been reported to impact foetal cardiac development 15. Lung disease may lead to right heart strain and pulmonary hypertension progressing to cor pulmonale 16. Conversely, cardiac dysfunction as measured by a reduced left ventricular ejection fraction may result in fluid overload causing pulmonary oedema and subsequent poor lung compliance. HIV has also been documented to cause primary pulmonary hypertension in adults 17 and adolescents 18.

The aim of this study was to investigate the prevalence and determinants of cardiopulmonary dysfunction in perinatally HIV‐positive adolescents on ART.

## 2 Methods

A prospective study, the Cape Town Adolescent Anti‐retroviral cohort (CTAAC), previously described 11, enrolled 515 perinatally HIV‐positive adolescents on ART and 110 age‐matched HIV‐uninfected adolescents. Patients were enrolled from August 2013 to April 2015 and followed six monthly at the Research Centre for Adolescent and Child Health at Red Cross War Memorial Children's Hospital, South Africa.

Participants were eligible for the study if they were adolescents aged 9 to 14 years, with perinatal HIV infection, had been on ART for at least six months and knew their HIV status. Informed parental consent and participant assent were obtained. Age‐matched HIV‐uninfected adolescents without known pre‐existing lung or cardiac disease were enrolled from Masiphumelele Youth Centre in Cape Town, South Africa. Perinatal HIV exposure of the HIV‐uninfected participants was unknown, but all tested negative for HIV prior to enrolment in the study. Ethical approval was obtained from the Human Research Ethics Committee of the University of Cape Town.

Data presented here are from the enrolment visit. Data on demography, self‐reported smoking history and cardiorespiratory symptoms, ART duration and adherence, previous pulmonary tuberculosis (PTB) and other lower respiratory tract infections (LRTI) were collected by validated questionnaires. A history of previous PTB or LRTI was extracted from hospital records and supplemented by participant or caregiver report. Respiratory symptoms of wheeze, shortness of breath were self‐reported. Blood was taken for CD4 count (Beckman Coulter^®^, Fullerton, CA, USA) and HIV viral load (Roche COBAS Ampliprep, Mannheim, Germany). Adherence to ART was self‐reported and measured using any missed doses in the last 30 days.

### 2.1 Lung function testing

Lung function testing 11 included spirometry measuring forced expiratory volume in one second (FEV_1_), forced vital capacity (FVC) and FEV_1_/FVC as measures of dynamic lung volumes and airflow obstruction; and the six‐minute walk test measuring effort tolerance. Lung function testing was deferred if the participant had an acute respiratory illness. Spirometry was done using the NDD Easyone Pro LAB (NDD, Zurich, Switzerland). All testing adhered to the American Thoracic Society/European Respiratory Society (ATS/ERS) guidelines 19, 20, 21. We reported the highest FVC or FEV_1_ from any of three acceptable spirometric attempts. The lower limit of normal (LLN) for spirometry outcome variables was calculated using the African American reference cohort in Global Lung Initiative (GLI) software, −1.64 standard deviations (SD) below the mean 22.

For the six‐minute walk test, the participant was instructed to walk for six minutes, between two marked cones placed 30 m apart, as per standardized recommendations 21. Heart rate, respiratory rate, blood pressure, Borg scale for dyspnoea and oxygen saturation were recorded before and at the end of testing. Distance covered in metres was recorded at the end of the test, with published reference data used as normative values 23.

Spirometry patterns were used to define abnormal lung function. Abnormal lung function was defined as abnormal spirometry with obstructive (FEV_1_/FVC less than the lower limit of normal (LLN), restrictive (FVC<LLN with normal FEV_1_/FVC) or mixed pattern spirometry (FEV_1_<LLN, FEV_1_/FVC<LLN and FVC<LLN).

### 2.2 Cardiac function testing

Cardiac function testing 24 was assessed by echocardiography, performed by a trained research echocardiographer using either a Philips IE33 or CX50 echo machines (Phillips, Netherlands) using standardized techniques 25, 26. All echocardiographs were interpreted by a single cardiologist. A random subset of 10% was also read by a second blinded cardiologist. Both cardiologists were blinded to the HIV status of the participant. Inter‐reader disagreements were resolved by consensus.

Right ventricular (RV) systolic function was determined by calculating the percentage fractional area change (FAC) and the volumetric RV ejection fraction that is, tricuspid annular plane systolic excursion (TAPSE). TAPSE was measured using M Mode echo 27 and FAC was measured by a two‐dimensional technique for tracing the area during systole and diastole by using the formula = (RV end‐diastolic area − RV end‐systolic area)/RV end‐diastolic area × 100 28. Pulmonary artery pressures (systolic and diastolic) were estimated using standard continuous and pulse wave Doppler methods. Cardiac dimensions were assessed in the standard manner either using direct measurement of 2‐D images or M Mode recordings. TAPSE is a global parameter for right ventricular function which describes apex‐to‐base shortening 29, 30. TAPSE has been found to be highly specific and easy method to estimate the right ventricular ejection fraction 31, 32. FAC has better correlation with cardiac MRI‐derived RV systolic dysfunction 33.

Left ventricular (LV) systolic function was determined by measuring shortening fraction (M‐mode) and deriving ejection fraction using the Teichholz method 34 and the modified Simpson's method 35. Left ventricular diastolic function was measured using Doppler assessment of mitral inflow. Tissue Doppler techniques were used to measure mitral annular velocity.

The following terminologies were used to define abnormal findings:

1. Right ventricular systolic dysfunction was defined as low TAPSE or low FAC. TAPSE *z*‐score <2 (*z*‐score was calculated based on published normal values) 36. *Z*‐scores were normalized to body surface area. 37. A fractional area change measurement of the RV (FAC) ≤34% was considered abnormal 38.
2. Pulmonary hypertension: Mean pulmonary arterial pressure (mPAP) was calculated using the Chemla equation. 2, 39 (mPAP = (0.61 × PAPs) + 2 mmHg) was normal if less than 25 mmHg 2.
3. LV systolic dysfunction: Left ventricular shortening fraction (LVSF) ≤25% 27.
4. LV diastolic dysfunction: E‐wave/A‐wave normal range was calculated according to age as per Eidem *et al*. 40.
5. Cardiopulmonary dysfunction was defined as any right ventricle or left ventricle systolic or diastolic dysfunction or abnormal mean pulmonary arterial pressure and abnormal spirometry or abnormal 6MWT

### 2.3 Data analysis

Descriptive statistics were used to describe characteristics of the study population and to summarize cardiopulmonary outcomes by HIV status. Comparison of cardiopulmonary and clinical outcomes by HIV status was compiled using the two‐sample test of proportions. Independent two‐sample *t*‐test was used to compare lung function in the adolescents with perinatally acquired HIV between those with low TAPSE and normal TAPSE. A new variable, cardiopulmonary status was generated; those with either obstructive or restrictive or mixed spirometry or abnormal 6MWT and any right or left systolic or diastolic dysfunction or abnormal mean pulmonary arterial pressure were considered as an impaired cardiopulmonary function. Univariate and multivariate logistic regression was done using the cardiopulmonary function as the outcome variable.

## 3 Results

**Figure 1:**
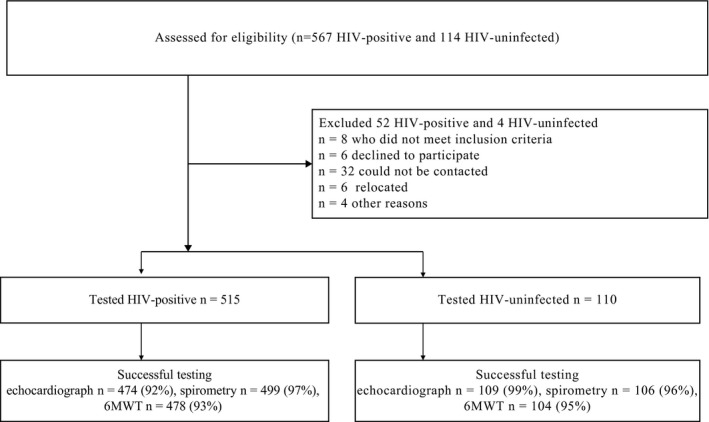
Flow diagram for the study population 6MWT, six‐minute walk test.

Five hundred and fifteen adolescents with perinatally acquired HIV and 110 HIV‐uninfected controls had lung function testing. Four hundred and seventy‐four adolescents with perinatally acquired HIV and 109 HIV‐uninfected completed echocardiogram testing; 478 HIV‐positive adolescents, 104 uninfected adolescents completed six‐minute walk test, Figure 1. Mean (SD) age was 12 (1.6) years and 50% were male. Median (IQR) duration of ART was 7.6 (4.6 to 9.3) years. Median (IQR) CD4 count was 712 (571 to 959) cells/mm^3^, 77.9% had viral load <50 copies/mL, Table 1. Sixty per cent of the participants were on two nucleoside‐reverse transcriptase inhibitors (NRTI), 75% on abacavir and one non‐nucleoside‐reverse transcriptase inhibitors (NNRTI), 98% were on efavirenz. Median age at ART initiation was 4.4 (2.0 to 7.0) years, with 29.7% initiating ART at <2 years of age. Cough and digital clubbing were more common in the adolescents with perinatally acquired HIV, *p* = 0.05 for both, Table 1. History of shortness of breath occurred rarely in less than 5% of participants, Table 1.

**Table 1:** Baseline characteristics of study population

Variable	HIV‐positive n = 474	HIV‐uninfected n = 109	*p* value*
Age, years	12.0 (1.6)	11.8 (1.8)	0.257
Male, n %	247 (51.7)	47 (45.2)	0.231
Height *z*‐score	−1.3 (1.1)	−0.5 (1.0)	<0.001
Respiratory rate, breaths/min	21.5 (3.5)	20.8 (5.1)	0.082
Viral load category, copies/mL		–	
<50, n (%)	369 (77.9)		
50 to 1000, n (%)	46 (9.7)		
1001 to 10,000, n (%)	32 (6.8)		
>10,000, n (%)	26 (5.5)		
CD4 count, cells/mm^3^	712 (571 to 959)	–	
WHO HIV staging at HIV diagnosis, n (%)			
I	34 (7.2)		
II	47 (9.9)		
III	266 (56.1)		
IV	105 (22.2)		
ART used			
NNRTI+2NRTI	282 (59.5)		
PI+2NRTI	175 (36.9)		
Others	9 (1.9)		
Previous PTB, n (%)	287 (58.5)	2 (0)	<0.001
Previous severe LRTI, n (%)	134 (28.3)	1 (0.9)	<0.001
Tobacco smoke exposure, n (%)	119 (25.0)	22 (20.9)	0.279
Poor ART adherence, n (%)	111 (23.4)	**–**	
ART duration, years, n (%)	7.0 (3.0)	**–**	
Age at ART initiation, years	4.4 (2.0 to 7.0)		
Shortness of breath, n (%)	16 (3.4)	2 (1.8)	0.402
History of wheeze, n (%)	51 (10.8)	6 (5.5)	0.096
History of cough, n (%)	69 (14.6)	8 (7.3)	0.045
History of doctor‐diagnosed asthma, n (%)	57 (12.0)	6 (5.5)	0.048
Finger clubbing, n (%)	16 (3.5)	0	0.052

ART, antiretroviral therapy; LRTI, lower respiratory tract infections; NNRTI, non‐nucleoside reverse transcriptase inhibitor; NRTI, nucleoside reverse transcriptase inhibitor; PI, protease inhibitor; PTB, pulmonary tuberculosis; WHO, World Health Organization.

Values are mean/SD except for age at ART initiation, viral load and CD4 which are median (IQR). *p values derived from chi‐square or two sample *t*‐test.

**Figure 2:**
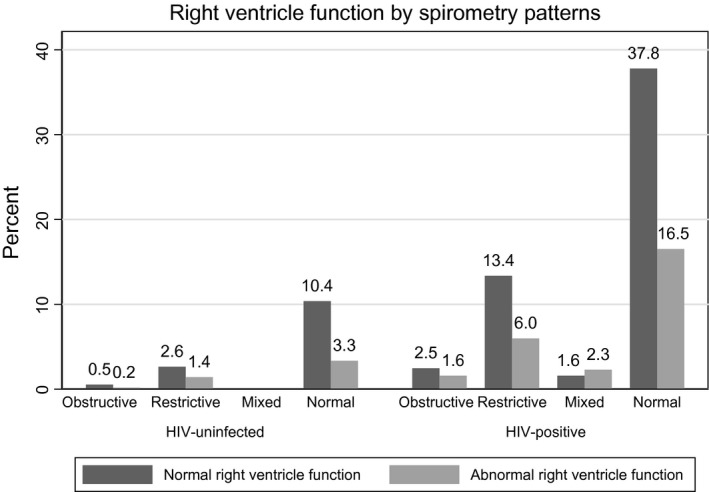
Spirometry pattern and right ventricle function

**Figure 3:**
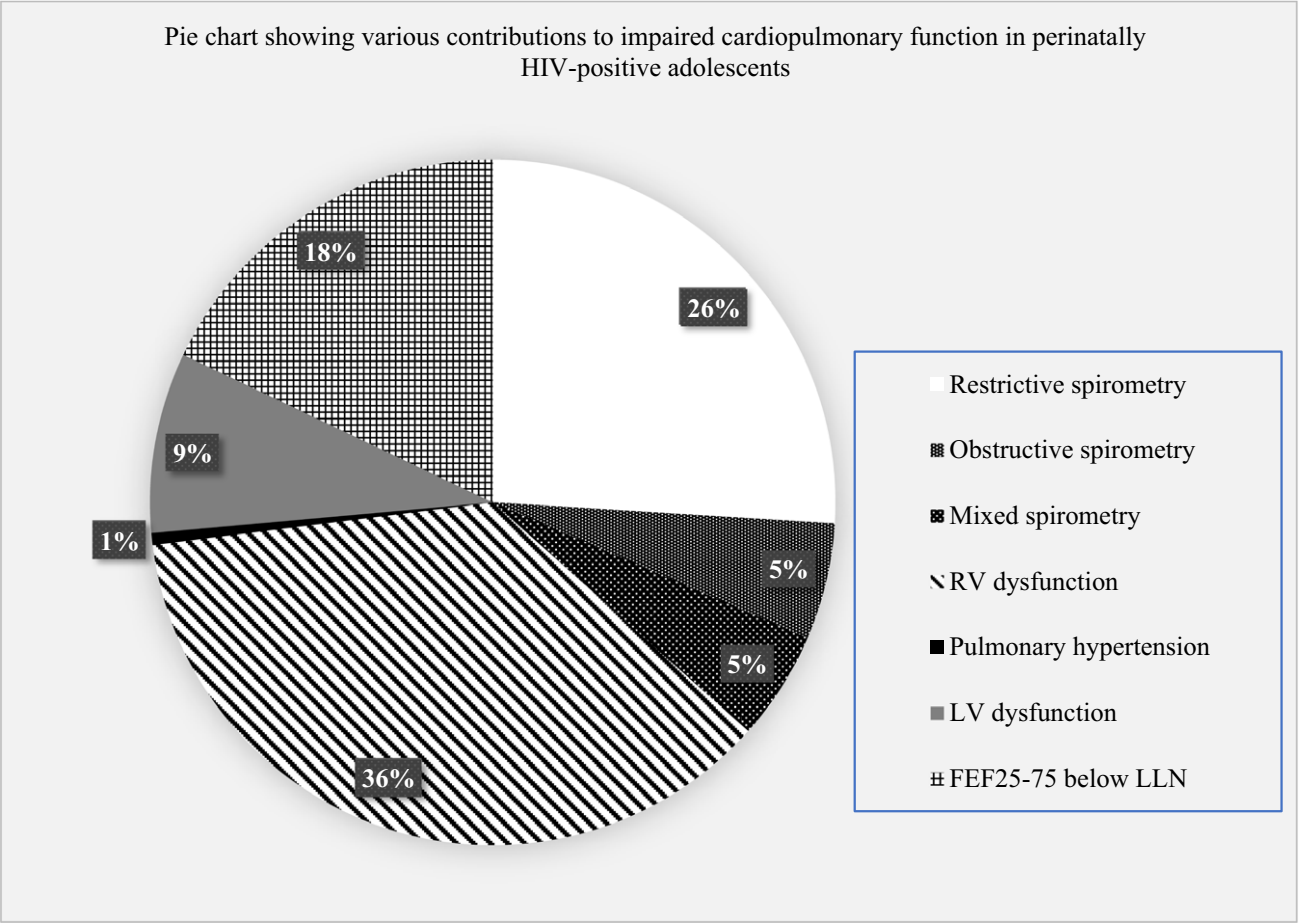
Lung and cardiac functional abnormalities in perinatally HIV‐positive adolescents FEF_25‐75_, forced expiratory flow at 25% and 75% of forced vital capacity; LLN, lower limit of normal calculated from African‐American reference values 22 **;** LV, left ventricle; RV, right ventricle.

Adolescents with perinatally acquired HIV with mixed pattern spirometry had a higher rate of RV dysfunction, Figure 2. None of the uninfected adolescents had mixed pattern spirometry. Obstructive and mixed pattern spirometry was reported in 5% of the HIV‐positive adolescents, Table 2. Right ventricle dysfunction (32%) was more common than left ventricular dysfunction (7%) in the adolescents with perinatally acquired HIV but not significantly different to uninfected adolescents (26.6% of whom had RV dysfunction and 5.5% LV dysfunction), Table 2. Thirteen per cent of perinatally HIV‐positive and 8.3% of uninfected adolescents had impaired cardiopulmonary function, Table 2. Sixteen per cent of HIV‐infected and 5% of uninfected adolescents had FEF_25 to 75_ less than the lower limit of normal for age, sex and height, Table 2. A pictorial diagram that shows the proportions of the various cardiac and lung function abnormalities in the HIV‐infected cohort is presented, Figure 3. Only two HIV ‐infected children had pulmonary hypertension, Table 2. Among adolescents with perinatally acquired HIV those with low TAPSE had significantly lower mean FEV_1_, 1.5 L versus 1.6 L, *p* = 0.011, Table 3.

**Table 2:** Cardiopulmonary measurements by HIV status

Variable, n (%)	n	HIV‐positive	n	HIV‐uninfected	*p* value*
Restrictive spirometry	474	110 (23.2)	109	23 (21.1)	0.637
Obstructive spirometry	474	23 (4.9)	109	4 (3.4)	0.596
Mixed pattern spirometry	474	22 (4.6)	109	0	0.022
FEF_25 to 75_ below LLN	474	76 (16.0)	109	6 (5.5%)	0.004
Right ventricular dysfunction (low TAPSE and low FAC)*	474	154 (32.5)	109	29 (26.6)	0.232
Pulmonary hypertension	474	2 (0.46)	109	0	0.476
Left ventricular diastolic dysfunction	474	36 (7.6)	109	6 (5.5)	0.447
Left ventricle systolic dysfunction	474	1 (0.2)	109	0	0.631
Cardiopulmonary function (impaired)*	474	64 (13.5)	109	9 (8.3)	0.136
Pulse before walk (mean/SD)	478	78.6 (12.5)	104	82.9 (14.2)	0.002
Pulse after walk (mean/SD)	478	82.9 (13.8)	104	87.1 (15.0)	0.004
Oxygen saturation before walk (mean/SD)	478	98.4 (2.0)	104	98.6 (0.8))	0.315
Oxygen saturation after walk (mean/SD)	478	98.3 (2.7)	104	98.4 (1.4)	0.675
MAP before 6MWT (mean/SD)	478	79.2 (7.7)	104	82.6 (8.1)	<0.001
MAP after 6MWT	478	83.0 (8.7)	104	86.6 (8.5)	0.001
Borg scale before 6MWT (mean/SD)	478	0.1 (0.2)	104	0.04 (0.2)	0.577
Borg scale after 6MWT (mean/SD)	478	1.3 (0.6)	104	1.3 (0.7)	0.737
Distance walked in 6 min (mean/SD)	478	437.8 (60.4)	104	443.8 (60.7)	0.380

6MWT, six‐minute walk test; FAC, fractional area change, Borg scale (perceived exertion scale); FEF_25 to 75_, forced expiratory flow at 25% and 75% of forced vital capacity; LLN, lower limit of normal calculated from African American reference values 22; MAP, mean arterial pressure (calculated from blood pressure); TAPSE, tricuspid annular plane systolic excursion.

* *p* value derived from chi‐square or two sample *t*‐test.

**Table 3:** Lung function by tricuspid annular plane systolic excursion (TAPSE) index in HIV‐positive adolescents

Lung function	n	Low TAPSE	n	Normal TAPSE	*p* value*
		Mean/SD		Mean/SD	
FEV_1_ (L)	114	1.5 (0.5)	350	1.6 (0.4)	0.011
FVC (L)	114	1.7 (0.5)	350	1.8 (0.5)	0.022
FEF_25 to 75_ (L)	114	2.0 (0.9)	350	2.2 (0.8)	0.041
FEV1/FVC	114	0.9 (0.1)	350	0.9 (0.1)	0.165

FEF_25 to 75_, forced expiratory flow at 25 to 75% of vital capacity; FEV_1_, forced expiratory volume in 1 sec; FVC, forced vital capacity; TAPSE, tricuspid annular plane systolic excursion.

* *p* value from two sample *t*‐test.

The cardiopulmonary functional status as measured by distance walked in six minutes, Borg scale and oxygen saturation were not different between the two groups, Table 2. Mean Borg scale was 1.3 in both groups, Table 2. Mean pulse rate and mean arterial pressures were lower in the adolescents with perinatally acquired HIV compared to the HIV‐uninfected, *p* < 0.05 for all, Table 2.

Height (OR 0.7, 95% CI 0.5 to 0.9), body mass index (OR 0.7, 95%CI 0.5 to 0.9) and past pulmonary tuberculosis (OR 2.3, 95%CI 1.2 to 4.4) were significantly associated with low cardiopulmonary function, Table 4. Those with digital clubbing had significantly higher odds for impaired cardiopulmonary function, OR 4.5, 95% CI 1.6 to 12.3, adjusted for age, sex, height and HIV status, Table 5.

**Table 4:** Associations of impaired cardiopulmonary function (n = 569) in perinatally HIV‐positive and uninfected adolescents

Variable	Univariate Odds ratio	*p* value*	95% CI	Multivariate Odds ratio	*p* value	95% CI
Age	1.1	0.365	0.9 to 1.2	–		
*z*‐height	0.8	0.017	0.6 to 0.9	0.7	0.010	0.5 to 0.9
*z*‐bmi	0.7	<0.001	0.5 to 0.8	0.7	0.009	0.5 to 0.9
ETS exposure	0.8	0.545	0.5 to 1.5	–		
Sex	0.8	0.419	0.5 to 1.3	–		
Past LRTI	1.6	0.124	0.9 to 2.7	1.4	0.321	0.7 to 2.5
Past PTB	2.1	0.013	1.2 to 3.9	2.3	0.017	1.2 to 4.4
Viral load copies/mL				–		
50 to 1000	0.8	0.677	0.3 to 2.2			
1001 to 10,000	1.9	0.171	0.8 to 4.6			
>10,000	1.7	0.331	0.6 to 4.6			
ART duration	1.1	0.062	1.0 to 1.2	1.1	0.228	0.9 to 1.2

ART, antiretroviral therapy; BMI, body mass index; ETS, environmental tobacco smoke; LRTI, lower respiratory tract infection; PTB, pulmonary tuberculosis.

* Adjusted for HIV status. Logistic regression.

**Table 5:** Association of respiratory symptoms/signs with impaired cardiopulmonary function (n = 569) in perinatally HIV‐positive and uninfected adolescents

Symptom	Univariate			Multivariate^b^		
	OR	95% CI	*p* value	OR	95% CI	*p* value
Wheeze	1.5	0.7 to 3.2	0.249	1.4	0.7 to 3.0	0.369
Shortness of breath	0.9	0.2 to 3.8	0.831	0.8	0.2 to 3.7	0.808
Cough	1.6	0.8 to 3.0	0.180	1.5	0.8 to 2.9	0.253
Digital clubbing	5.2	1.9 to 14.0	0.001	4.5	1.6 to 12.3	0.004
Asthma^a^	1.5	0.7 to 3.2	0.249	1.3	0.6 to 2.7	0.445

Symptoms are self‐reported except digital clubbing ^a^physician‐diagnosed asthma; ^b^adjusted for HIV status, age, sex, height. Logistic regression.

## 4 Discussion

This study provides comprehensive lung function and cardiac function data on a large cohort of perinatally HIV‐positive South African adolescents on ART showing that a proportion of HIV‐positive adolescents had cardiopulmonary dysfunction despite being on long‐term ART and having well‐controlled HIV disease. However, the prevalence of cardiopulmonary dysfunction was similar to the uninfected adolescents. This may be due to lack of validated local African reference values for cardiac function parameters or measurement error in the echocardiography measurements.

Low FEF_25 to 75_, a marker of small airways disease, was significantly higher in the HIV‐positive group, consistent with studies of HIV‐positive adolescents in sub‐Saharan Africa that have reported a predominance of small airways lung disease 6, 41. However, we did not include low FEF_25 to 75_ in the definition of impaired cardiopulmonary function but used FEV_1_ or FVC as the standard measurement of clinically relevant lung disease 42.

Height, body mass index and previous history of pulmonary tuberculosis were associated with impaired cardiopulmonary function; and digital clubbing had higher odds of low cardiopulmonary function. Those with low TAPSE had lower lung function.

Contrary to the few published studies 43, 44, 45 that showed reduced exercise capacity as measured by treadmill or six‐minute walk test in HIV‐positive adolescents, this study did not show any differences in distance walked nor oxygen saturation post‐exercise between perinatally HIV‐positive and uninfected adolescents. This may reflect differences in study populations, as in the current study, the perinatally HIV‐positive adolescents were relatively well established on ART and had good control of HIV disease. It may also be due to differences in the exercise test modalities or outcome measured; the current study reported distance walked and oxygen saturation before and after the test, while the outcome measure was peak oxygen consumption (VO2max) in other studies 44, 45 43. Furthermore, the six‐minute walk test is an insensitive measure of mild cardiopulmonary compromise. It is a submaximal test and less predictive of VO2 max compared to the shuttle‐walk test 46. The lower mean heart rate and arterial pressure observed in adolescents with perinatally acquired HIV requires further study.

Right ventricular dysfunction was more common than left ventricular dysfunction in these data. However, this finding must be interpreted cautiously as the normal reference ranges used were from North America and symptoms of RV dysfunction in this cohort were minimal. Miller *et al*. 4 in a Zimbabwe cohort reported 29% of right ventricular dysfunction in a cohort that had a high rate of abnormal lung function 6. The prevalence of restrictive spirometry pattern in this cohort, Table 2, was unexpected and requires further exploration in an ongoing longitudinal study.

The finding that body mass index, height and past pulmonary tuberculosis were associated with impaired cardiopulmonary dysfunction may reflect the fact that stunting and opportunistic infections affect lung function and may lead to subsequent cardiac dysfunction. Pulmonary TB has also been associated with chronic obstructive pulmonary disease in adults with HIV 47. Previous data 11 reported that PTB and previous pneumonia were more prevalent in HIV‐positive adolescents and were both associated with low lung function. In addition, the higher likelihood of impaired cardiopulmonary dysfunction in those with digital clubbing may reflect lung disease leading to consequent heart dysfunction. Digital clubbing is well known to be associated with chronic cardiac or lung disease 48. Similarly, nail clubbing was common in a Malawi cohort of HIV‐positive adolescents with a high prevalence of chronic lung disease 49.

The strengths of this study include a large sample size of perinatally HIV‐positive adolescents on ART with a comparative group of HIV‐uninfected adolescents and the comprehensive lung and cardiac function tests. Our study was limited by the independent assessment of lung and cardiac function rather than a combined measure of cardiopulmonary function such as formal cardiopulmonary exercise testing. This would measure oxygen consumption and carbon dioxide excretion by sampling inspired and expired gas during exercise 50. Such technology is expensive and inaccessible in most paediatric centres in South Africa. However, we used a broader definition of impaired cardiopulmonary function to include both heart and lung function parameters used in our study. The cardiac function measures, TAPSE and FAC, are limited by the absence of African reference parameters; normative values were derived from a Caucasian population. Nevertheless, the uninfected control group served as a comparator.

## 5 Conclusions

This study indicates that cardiopulmonary dysfunction occurs in an appreciable proportion of African adolescents with perinatally acquired HIV despite being on ART and having well‐controlled HIV disease. However, the prevalence of cardiopulmonary dysfunction was similar in HIV‐uninfected adolescents, which may reflect lack of validated local African reference values for echocardiography measurements. In turn, this study highlights the need for development of more sensitive markers of lung or heart disease in adolescents with perinatally acquired HIV, given the minimal symptoms that participants reported. The study identified risk factors for cardiopulmonary dysfunction such as prior PTB and impaired nutrition highlighting areas that may be amenable to public health interventions to optimize health.

## Competing interests

None.

## Authors’ contributions

LG: data collection, statistical analysis and wrote the manuscript.

SM: analysed cardiac function data and wrote the section on cardiac function testing.

LZ: read all the echocardiography, critically reviewed the manuscript and provided comments which were incorporated into this manuscript.

JL: advised on data analysis, critically reviewed the manuscript and provided comments which were incorporated into this manuscript.

DG: data collection, critically reviewed the manuscript and provided comments which were incorporated into this manuscript.

LM: conception and designing of study, obtained funding, advised on data analysis, critically reviewed the manuscript and provided comments which were incorporated into this manuscript.

HZ: conception and designing of study, obtained funding, advised on data analysis and wrote the manuscript.
